# Multiregional Sequencing of IDH-WT Glioblastoma Reveals High Genetic Heterogeneity and a Dynamic Evolutionary History

**DOI:** 10.3390/cancers13092044

**Published:** 2021-04-23

**Authors:** Sara Franceschi, Prospero Civita, Francesco Pasqualetti, Francesca Lessi, Martina Modena, Serena Barachini, Mariangela Morelli, Orazio Santonocito, Riccardo Vannozzi, Geoffrey J. Pilkington, Valerio Ortenzi, Antonio Giuseppe Naccarato, Paolo Aretini, Chiara Maria Mazzanti

**Affiliations:** 1 Fondazione Pisana per la Scienza, 56017 Pisa, Italy; f.lessi@fpscience.it (F.L.); m.modena@fpscience.it (M.M.); serena.barachini@med.unipi.it (S.B.); m.morelli@fpscience.it (M.M.); p.aretini@fpscience.it (P.A.); c.mazzanti@fpscience.it (C.M.M.); 2 School of Pharmacy and Pharmaceutical Sciences, College of Biomedical and Life Sciences, Cardiff University, Cardiff CF10 3NB, UK; prospero.civita@port.ac.uk (P.C.); geoff.pilkington@port.ac.uk (G.J.P.); 3 Brain Tumour Research Centre, School of Pharmacy & Biomedical Sciences, University of Portsmouth, Portsmouth PO1 2DT, UK; 4 Department of Radiation Oncology, Azienda Ospedaliera Universitaria Pisana, University of Pisa, 56126 Pisa, Italy; f.pasqualetti@ao-pisa.toscana.it; 5 Institute of Life Sciences, Scuola Superiore Sant’Anna, 56127 Pisa, Italy; 6 Division of Neurosurgery, Spedali Riuniti di Livorno—USL Toscana Nord-Ovest, 57124 Livorno, Italy; orazio.santonocito@uslnordovest.toscana.it; 7 Department of Neurosurgery, University of Pisa, 56126 Pisa, Italy; r.vannozzi@ao-pisa.toscana.it; 8 Department of Basic and Clinical Neuroscience, Division of Neuroscience, Institute of Psychiatry & Neurology, King’s College London, London SE5 9RX, UK; 9 Department of Translational Research and New Technologies in Medicine and Surgery, Division of Surgical Pathology, University of Pisa, 56126 Pisa, Italy; valerio.ortenzi@ao-pisa.toscana.it (V.O.); giuseppe.naccarato@unipi.it (A.G.N.)

**Keywords:** glioblastoma, multiregional sequencing, spatial heterogeneity, temporal heterogeneity, tumor progression, clonal evolution, tumor phylogeny

## Abstract

**Simple Summary:**

Glioblastoma is the most common and aggressive primary brain malignancy in adults. In addition to extensive inter-patient heterogeneity, glioblastoma shows intra-tumor extensive cellular and molecular heterogeneity, both spatially and temporally. This heterogeneity is one of the main reasons for the poor prognosis and overall survival. Moreover, it raises the important question of whether the molecular characterization of a single biopsy sample, as performed in standard diagnostics, actually represents the entire lesion. In this study, we sequenced the whole exome of nine spatially different cancer regions of three primary glioblastomas. We characterized their mutational profiles and copy number alterations, with implications for our understanding of tumor biology in relation to clonal architecture and evolutionary dynamics, as well as therapeutically relevant alterations.

**Abstract:**

Glioblastoma is one of the most common and lethal primary neoplasms of the brain. Patient survival has not improved significantly over the past three decades and the patient median survival is just over one year. Tumor heterogeneity is thought to be a major determinant of therapeutic failure and a major reason for poor overall survival. This work aims to comprehensively define intra- and inter-tumor heterogeneity by mapping the genomic and mutational landscape of multiple areas of three primary IDH wild-type (IDH-WT) glioblastomas. Using whole exome sequencing, we explored how copy number variation, chromosomal and single loci amplifications/deletions, and mutational burden are spatially distributed across nine different tumor regions. The results show that all tumors exhibit a different signature despite the same diagnosis. Above all, a high inter-tumor heterogeneity emerges. The evolutionary dynamics of all identified mutations within each region underline the questionable value of a single biopsy and thus the therapeutic approach for the patient. Multiregional collection and subsequent sequencing are essential to try to address the clinical challenge of precision medicine. Especially in glioblastoma, this approach could provide powerful support to pathologists and oncologists in evaluating the diagnosis and defining the best treatment option.

## 1. Introduction

Glioblastoma (GB) is the most common and aggressive primary brain malignancy in adults and one of the deadliest human cancers [1,2,3]. Mean survival rates are around 14 months with current and aggressive therapeutic modalities that include maximal safe, surgical resection, followed by radiotherapy and concomitant and adjuvant chemotherapy with the alkylating agent temozolomide [2,3,4,5]. Although GB is a rare cancer with an overall incidence of less than 10 per 100,000 people, its poor prognosis makes it a crucial public health problem [6]. Malignant gliomas are the cause of 2.5% of cancer deaths and are the third leading cause of cancer death in people between 15 and 34 years of age [7]. Glioblastomas are divided in the 2016 World Health Organization’s WHO Classification of Tumors of the Central Nervous System [5,8] into two groups, based on genetic mutations in isocitrate dehydrogenase genes (IDH1 and IDH2): IDH-WT GB (90% of cases), defined as primary or de novo glioblastoma, and IDH-mutant GB, called secondary glioblastoma, with a history of previous lower-grade diffuse glioma. Overall survival (OS) in IDH1-mutant GB is more than three times higher than in IDH-WT GB [9].

The impossibility of a total tumor debulking and a poor distribution of drugs in the brain, due to the presence of the blood-brain barrier (BBB), contribute significantly to the lack of effective treatment options and a poor prognosis [1,3]. However, clinical results vary considerably between patients. Previous studies have shown marked differences between tumors at the genomic and transcriptomic level, which may be the basis for differences both in a patient’s natural tumor history and in treatment responses [2]. In addition to inter-patient differences, GB evolution results in spatial and temporal intra-tumor heterogeneity [1,10].

Tumor heterogeneity is one of the main reasons for the poor prognosis and overall survival due to therapeutic failure and drug-resistance [1,3,11,12,13]; therefore, it represents a challenge to achieve the goals of precision medicine. This raises the important question of whether the molecular characterization of a single portion of the tumor sufficiently represents the genomic landscape of GB in a biologically and clinically significant way for personalized-medicine approaches. Therapy selection based on the analysis from a single biopsy specimen may not be representative of the entire lesion and could result in treatment failure [2,14,15].

To face these challenges and fully define the mutational landscape and intra-tumor heterogeneity of GBs at the patient level, we sequenced the whole-exome of nine spatially different cancer regions of three primary IDH-WT GBs. Our results show that tumor heterogeneity displays a specific signature that reveals the evolutionary dynamics of GB at the individual patient level, highlighting the need for multiregional sequencing for precise therapy.

## 2. Materials and Methods

### 2.1. GB Patients

Three primary human GB surgical specimens (GB01, GB02 and GB03), diagnosed according to WHO diagnostic criteria [8], were collected from the Department of Neurosurgery (University of Pisa). Tumors were resected by the same surgeon and reviewed by the same pathologist. Patients received similar medical treatments. All cases had a diagnosis of GB with no previous history of any brain neoplasia and were not carrying R132 IDH1 or R172 IDH2 mutations or the 1p/19q codeletion. Patient clinical and demographic data are shown in Table 1.

The study was approved by the Ethics Committee of the University Hospital of Pisa and all methods were performed in accordance with approved guidelines. Patients’ data and samples were completely anonymized.

### 2.2. Sample Collection

**Figure 1:**
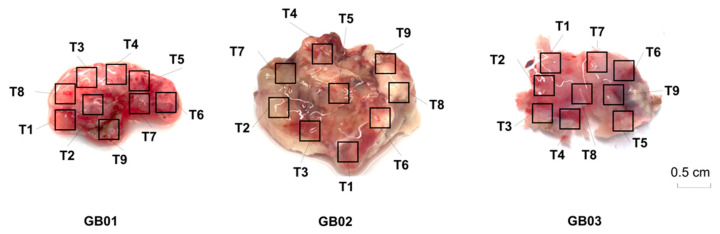
Multiregional sampling performed on resected tumors. Nine spatially separated regions of approximately 3 mm^3^ were collected from each primary tumor.

All surgically resected tumor samples were chosen by the neurosurgeon during surgery. Spatially separated tumor regions, documented by photography, were cut with a scalpel and collected immediately after surgery under a biological hood. On the surface of each tumor, nine spatially organized samples (T1–T9) of approximately 3 mm^3^ each, were collected with locations as shown in Figure 1.

Single portions were then snap-frozen in liquid nitrogen for subsequent DNA extraction.

### 2.3. Sample Processing

Each tumor region was used for genomic DNA extraction using a modification of the Maxwell 16 FFPE Tissue LEV DNA Purification Kit (Promega, Madison, WI, USA). Specifically, incubation with Proteinase K (Promega, Madison, WI, USA) and Incubation Buffer (Promega, Madison, WI, USA) was performed for one hour at 56 °C. DNA concentration was determined using the Qubit Fluorometer (Life Technologies, Carlsbad, CA, USA) and the quality was assessed using the Agilent 2200 Tapestation (Agilent Technologies, Santa Clara, CA, USA) system.

### 2.4. Multi-Region Whole-Exome Sequence (WES) Analysis

For each tumor portion, an exome library was prepared from 50 ng DNA using Nextera Exome Kit (Illumina, San Diego, CA, USA) following the manufacturer’s instructions. Each NGS run included 9 pooled libraries loaded into one NextSeq High Output cartridge (300 Cycles; Illumina). Paired-end sequencing was performed on a NextSeq 500 system (Illumina) with 151 bp sequencing.

### 2.5. Bioinformatic Analysis and Data Interpretation

Quality control was performed on fastq files using FastQC (v011.9) and FastqScreen (v0.14.1) [16]. Paired-end reads were aligned to GRCh37/hg19 using BWA-MEM [17] aligner algorithm with default parameters. Mapped reads were sorted with SMA and MarkDuplicates tools (http://broadinstitute.github.io/picard, accessed on 16 March 2020). Target coverage of the exome sequencing of all analyzed samples averaged 70.4X. Aligned reads were processed using GATK [18] to remove low mapping quality reads (MPQ ≥ 20) and realigned in the genomic regions around potential indels. Base quality scores were recalibrated for the BAM files using GATK.

Somatic single-nucleotide variants (SNVs) and indels were identified in tumors against a PoN (Panel of Normal, GATK best practices, https://console.cloud.google.com/storage/browser/gatk-best-practices/somatic-b37, accessed on 16 March 2020) by using Mutect2 [19] variant calling algorithms. Rare variants were obtained excluding somatic variants reported in the non-cancer database gnomAD v3 [20] setting minor allele frequency (MAF) of ≥0.01. The frequency and type of mutations were investigated using the R package MAFtools [21].

Copy number was estimated by CNVkit [22]. Copy number variations were summarized using CNApp [23] with default cutoffs. Comparison data for CNV classifier were downloaded from The Cancer Genome Atlas Glioblastoma Multiforme (TCGA-GB, https://www.cancer.gov/tcga, accessed on 16 March 2020) data collection (hg19 Legacy Database) using the TCGAbiolinks [24] and randomForest [25] R packages.

## 3. Results

### 3.1. Whole-Exome Sequencing

A total of 27 intra-tumor regions, derived from three different human primary GBs, were subjected to whole exome sequencing (WES). Data analysis showed in coding regions an average of more than 33, 31 and 29 million of mapping reads, in GB01, GB02 and GB03, respectively. Total mean coverage was 70.36X (SD = 2.39). Further information on the run is shown in Table S1. Nucleic acid sequence data are available from The European Genome-phenome Archive (EGA): https://ega-archive.org, accessed on 8 March 2021) (submission ID: EGAD00001007063).

### 3.2. Copy Number Variation Analysis

**Figure 2:**
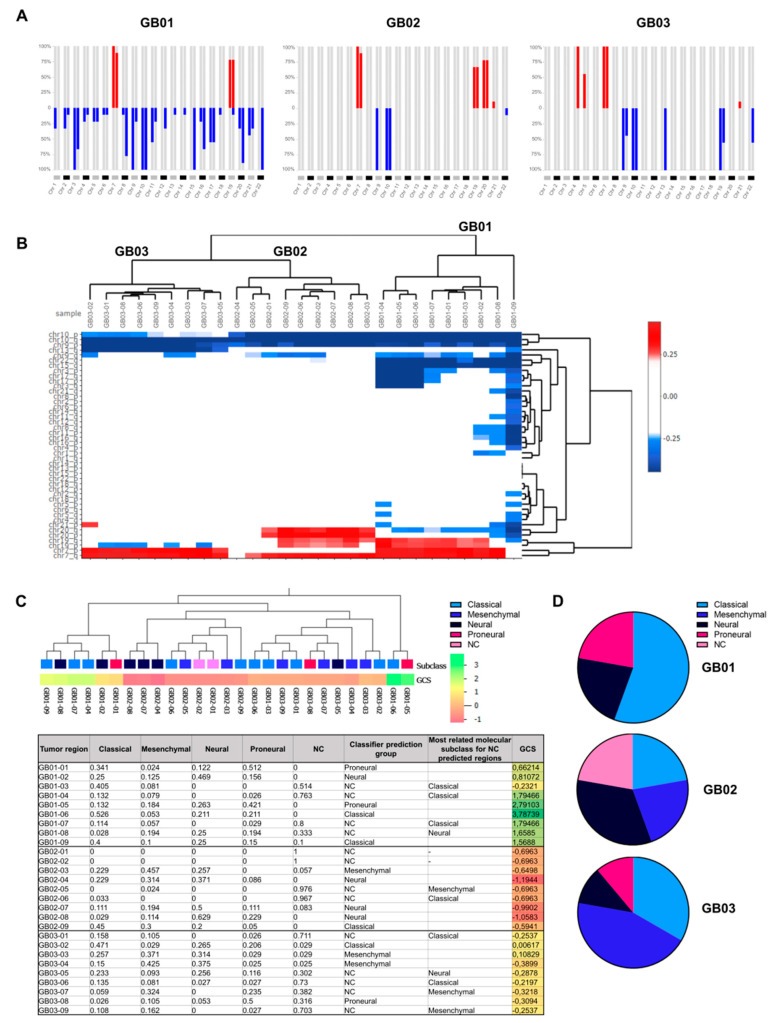
Copy number variation (CNV) analysis and molecular subtype correlation. (**A**) CNApp frequencies for the p and q arms of each chromosome. Alteration frequency is expressed as the percentage of altered regions out of the total of 9 regions within each tumor (red for gains and blue for losses). (**B**) Hierarchical clustering of copy number variations of chromosomal regions (p and q arms). Hierarchical cluster analysis according to Pearson’s correlation of the three GB samples subdivided into their intra-tumoral regions. (**C**) Correlation with the four molecular subtypes and hierarchical clustering using the random forests algorithm. The CNApp classifier model was applied to our three GBs and 480 GBs derived from the TCGA-GBM data collection with molecular subclass annotation. Tumor regions in our 3 GBs were included in the classifier as “not classified” (NC) and correlation was performed using the global score (GCS) that CNApp assigns during resegmentation, by which it classifies and weights CNVs based on their length and width. The values in the table are the correlation coefficients that each region has with each of the four molecular subclasses and with the NC class. The “Classifier prediction group” column reports the molecular subtype that the system has correlated and predicted. The “Most related molecular subclass for NC predicted regions” column reports the best correlation found with one of the four molecular subclasses when the tumor region was associated with NC by the classifier. (**D**) Pie chart of the molecular composition of each tumor. Each tumor is composed of several molecular subclasses, each associated with an intra-tumoral region.

Copy number variation (CNV) analysis was carried out as a first analysis to provide an overview of the chromosomal structural abnormalities and genomic instability, both in the whole tumor and in the single intra-tumor portions. Through CNApp [23] we calculated the chromosomal alterations of the entire tumor (Figure 2A).

As shown in Figure 2A the alteration (gain or loss) of the short (p) and long (q) arm of each chromosome is displayed as a percentage variable from 0 to 100%. This percentage represents the number of tumor portions out of the total (nine portions) that are affected by the specific alteration (Figure 2A). In addition, with the CNApp, we also clustered the 27 intra-tumor regions (Figure 2B), to identify the differences and similarities in the copy number variations among regions of the same tumor and different tumors. The CNV analysis revealed a considerable aneuploidy with whole-arm and whole-chromosome alterations (gains and losses), demonstrating chromosomal instability across our samples (Figure 2A,B). The most evident chromosome alterations present in all three GB samples are chromosome 9 and 10 deletion and chromosome seven amplification. The long arm of chromosome 10 has major losses in all 27 tumor regions (Figure 2A,B). The short arm of chromosome 10 is deeply deleted in all regions of GB01 and GB02 while it has a smaller deletion in the GB03 portions (Figure 2A,B). 9p is also deleted in all 27 tumor regions, whereas 9q has deletions in only few regions (eight/nine in GB01, five/nine in GB02, and three/nine in GB03) (Figure 2A,B). All regions, except regions 04 of GB02 and 09 of GB01, have 7q amplification (Figure 2B). The short arm of chromosome 7 is amplified in GB03 (all 9 regions) and GB01 (all regions except region 09), whereas it has no alterations in GB02 regions (Figure 2B). Other common chromosomal alterations observed in the three different tumors are 13q deletion (all regions of GB03 and region 09 of GB01) and 19q amplification (seven/nine and six/nine regions of GB01 and GB02, respectively) (Figure 2B). GB03 shows loss of the 19p arm, whereas in some regions of GB01 and GB02 are amplified (Figure 2A,B). GB01 has a loss of 20q while in GB02 shows a gain of the entire chromosome (Figure 2A,B). GB01 displays increased numbers of altered DNA copies, especially in chromosomes 3, 8, 15, 16, and 22 (Figure 2A,B). Notably, all regions of GB01 share common alterations in chromosomes 15 and 22, with a deletion of the long arm (Figure 2A,B). Three out of nine regions of GB01 (GB01-04/05/06) have, on chromosomes 3 and 17, common gene copy number alterations distributed throughout the chromosomes (Figure 2B). On chromosomes 8 and 16, one/nine regions of GB01 (GB01-09) has exclusive gene copy number deletions located on both the long and short arms (Figure 2B).

Genome-wide DNA copy number alteration profiles have previously been correlated with the four molecular subtypes of GB: Classical, Mesenchymal, Neural, and Proneural, according to the study initiated by Phillips et al. [26] and the subsequent Verhaak classification [27]. This classification is based on fourteen amplifications and seven homozygous or hemizygous deletion events, both broad and focal, on chromosomes 4 (4q12); 7 (7p11.2, 7q21.2, 7q31.2 and 7q34); 9 (9p21.3); 10 (10q23); 13 (13q14) and 17 (17q11.2). Considering the aforementioned chromosome alterations, we used the CNApp classifier [23] to calculate the global score (GCS) that the system assigns during re-segmentation by classifying and weighting CNVs according to their length and width. Next, the GCS was used to correlate the 27 intra-tumor regions with the 480 GBs from the TCGA-GB data collection (https://www.cancer.gov/tcga, accessed on 4 December 2020), to determine which GB molecular subclass they belonged to or resembled the most. The correlation analysis is shown in Figure 2C, which consists of a clustering dendrogram of our 27 samples based on their GCSs (upper section) and a table with the correlation coefficients related to the four molecular subclasses and CGS values (lower section). Based on the correlation coefficients, the classifier assigns a molecular subclass (third-to-last column, Classifier prediction group, Figure 2C). In cases where the system was unable to assign a sample to any of the four molecular subclasses, we manually associated the sample to the class with the highest correlation coefficient (second-to-last column, Most related molecular subclass for NC predicted regions, Figure 2C). The analysis revealed a highly variable CGS (−1.3 to 3.8) both among the three tumors and among regions of the same tumor, further confirming the existence of distinct CNV profiles (last column, Figure 2C). Fourteen out of twenty-seven regions were correlated by the classifier to one of the four GB molecular subclasses (Figure 2C). Eleven out of thirteen regions, not automatically correlated by the system, were manually associated with the molecular class for which they had the highest correlation coefficient (Figure 2C). Two regions of GB02 (01 and 02) were not correlated by the classifier or manually associated with a specific molecular subtype because they had no correlation with any of the four molecular subclasses (correlation coefficient 0) (Figure 2C). GB01 was mostly assigned to the Classical subtype (five/nine regions), while three/nine regions of GB02 are associated with the Neural subclass. GB03 was mostly assigned to the Mesenchymal subclass (four/nine) (Figure 2D). Nevertheless, for all three samples, within the same tumor, we found different molecular subtypes (Figure 2D).

### 3.3. Mutational Landscape

Initially we analyzed the genomic alterations by selecting all mutations in the coding region (known to alter protein function), as well as all splicing mutations, excluding synonymous mutations that had no predicted impact on splicing. We discovered a total of 97,732, 50,562 and 35,498 variants for tumor GB01, GB02 and GB03, respectively.

**Figure 3:**
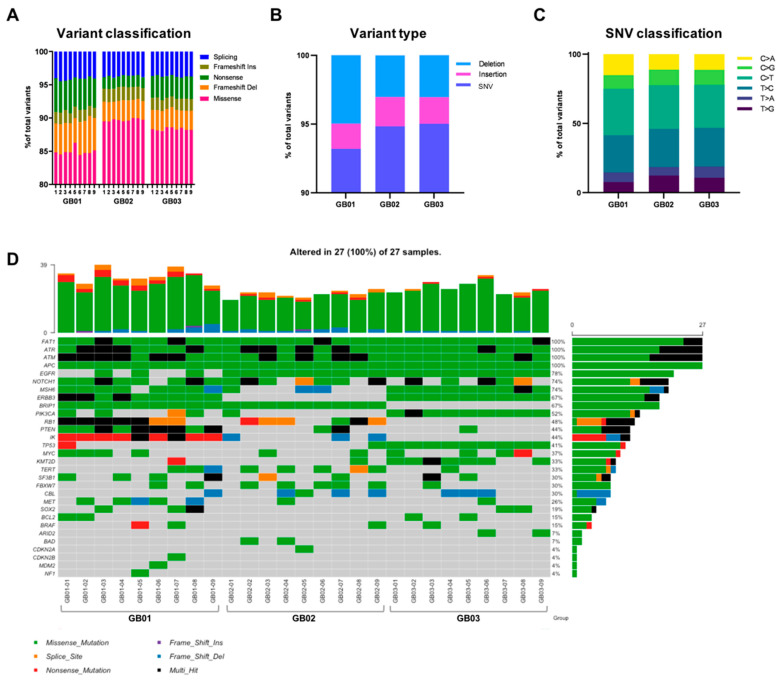
Mutation spectrum in GB. (**A**) Percentage of variant classification, plotted as a percentage of the total number of variants detected. (**B**) Percentage of mutation type, plotted as a percentage of the total number of variants detected. (**C**) Percentage of single-nucleotide variant (SNV) classification, plotted as a percentage of the total number of SNVs detected. (**D**) Oncoplot of the distribution of mutations found in our samples in the most frequently mutated genes in GB. Each column represents one sample (9 regions per tumor) and each row a different gene. Colored squares show mutated genes, while empty (gray) squares show no mutated genes. The different types of mutations are colored according to the type of variant: orange, splice site mutation; blue, frameshift deletion; green, missense mutation; red, nonsense mutation; and black, multi-hit mutation. Genes annotated as “multi-hit” have more than one type of mutation in the same region. The bar graph on the right shows the total number and percentage of mutated regions for each gene, out of the total 27 regions, colored according to mutation type. The upper graph shows the total number and type (different color) of mutations for each tumor region.

The variant classification plot (Figure 3A), shows the number and classification of the variants in each tumor region, providing percentages of missense, nonsense, splice-site, and frame shift mutations (both insertion and deletion) out of the total number of mutations.

GB01 has fewer missense mutations overall than GB02 and GB03, counting more frameshift deletions and nonsense mutations. In Figure 3B, mutations are classified by type (single nucleotide variants-SNVs, deletion, and insertion), represented as a percentage of the total in the whole tumor. Again, the mutation type classification of GB01 differs from the other two tumors, having fewer SNVs and more deletions than GB02 and GB03, which instead maintain a more similar profile to each other (Figure 3B). Specifically, we observed 96,640 SNVs, 914 insertions and 375 deletions in GB01; 48,050 SNVs, 992 insertions and 1621 deletions in GB02; and 33,864 SNVs, 233 insertions and 1446 deletions in GB03 (Figure 3B). Within SNVs we find a similar percentage in the substitution of T > C, C > T, and C > G alleles among the three samples (Figure 3C). GB02 has a higher percentage of T > G, GB03 of T > A and GB01 of C > A compared to the other tumor samples (Figure 3C).

### 3.4. Somatic Mutations

Somatic mutations were called using Mutect2 [19]. We started by focusing on the genes known to be altered in GB IDH1-WT, referring to two databases: my cancer genome–MCG, mycancergenome.org and American Brain Tumor Association (ABTA), abta.org. We found 30 mutated genes in the three GB samples from a total of 264 different variants. The oncoplot in Figure 3D represents each of the 30 genes: the type(s) of gene mutation present in each of the 27 intra-tumor regions (colored square, middle section of the graph), the percentage of tumor regions out of the total (27) reporting the specific gene mutation(s) (bar graph on the right) and the total number of variants, considering all 30 genes, for each specific tumor region (bar graph at the top). Some genes (*APC*, *ATM*, *ATR*, and *FAT1*) are mutated in all 27 regions of the three GB samples (Figure 3D). In particular, *ATM*, *ATR*, and *FAT1* show multi-hit mutations (>2 types of mutations on the same gene, black squares, Figure 3D). *EGFR* carries mutations in all tumor regions of GB02 and GB03 while only three regions of GB01 have *EGFR* mutations (Figure 3D). *ERBB3* has mutations in all tumor regions of GB01 and GB03, whereas GB02 has no *ERBB3* mutations in any of its regions (Figure 3D). All regions of GB01 and GB02 share the mutated *BRIP1* gene, whereas all regions of GB03 demonstrate no mutations in *BRIP1* (Figure 3D). GB01 has mutations in *PTEN* and *IK* in all nine regions, as well as GB03 has all regions with mutations in *MSH6*, *PIK3CA* and *TP53* (Figure 3D). There are also several genes that carry mutations in only one or two of the nine regions within the same tumor, such as *ARID2*, *BAD*, *BCL2*, *BRAF*, *CBL*, *CDKN2B*, *FBXW7*, *KMT2D*, *MDM2*, *MET*, *MYC*, *PIK3CA*, *PTEN*, *SF3B1*, *SOX2*, *TERT* and *TP53* (Figure 3D).

To establish the clinical significance of the 264 somatic variants found, we used Varsome [28] a search engine for human genomic variations. The pathogenicity of variants is reported using an automated classifier that evaluates the variant according to American College of Medical Genetics and Genomics (ACMG) guidelines [29], classifying it as one of the following: “pathogenic”, “probably pathogenic”, “probably benign”, “benign”, or “uncertain significance”. A total of 59 variants in 18 genes were identified as clinically relevant by being classified as pathogenic or likely pathogenic (Table 2): *APC* (*n* = 1), *ATM* (*n* = 2), *ATR* (*n* = 3), *BRAF* (*n* = 2), *EGFR* (*n* = 2), *ERBB3* (*n* = 3), *KMT2D* (*n* = 2), *MET* (*n* = 2), *MSH6* (*n* = 3), MYC (*n* = 1), *NOTCH1* (*n* = 6), *PIK3CA* (*n* = 7), *PTEN* (*n* = 4), *RB1* (*n* = 10), *SF3B1* (*n* = 5), *SOX2* (*n* = 2), *TERT* (*n* = 2) and *TP53* (*n* = 2). All of these 59 variants are heterozygous mutations and include frameshift (*n* = 13), missense (*n* = 23), nonsense (*n* = 12) and splice site (*n* = 11) mutations (Table 2).

None of these mutations are shared by all regions of the three tumors, but there are mutations that are common among one or more regions of different tumors (Table 2): *ATM*^Ser421Ter^ shared by GB01 (regions 01–04, 06 and 07) and GB02 (regions 03, 07 and 08); *MSH6*^Phe1088SerfsTer2^ in common between GB01 (region 09); GB02 (regions 05 and 06) and GB03 (region 08) and *RB1*^Ser474Ile^ present in GB01 (regions 01–07) and GB02 (regions 03, 04, 08 and 09) and *RB1*^c.1421 + 1G > T^ present in GB01 (regions 02, 03 and 05) and GB02 (region 03). Most of the variants (51/59) are mutations affecting only one region of a tumor (Table 2). Among the 59 variants, 26 variants are mutations noted in COSMIC, and among them, two variants have already been described in brain pathology in COSMIC (Table 2): *EGFR*^Arg108Lys^ present in all regions of GB02 and *KMT2D*^Arg2830Ter^ in region 07 of GB01.

### 3.5. Rare Variants

In order to differentiate our somatic mutations as common or rare variants in the population, we set a minor allele frequency (MAF) threshold of less than 0.01 to define rare variants. We discovered a total of 17,068, 4255, and 3039 rare variants for GB01, GB02, and GB03 tumors, respectively.

**Figure 4:**
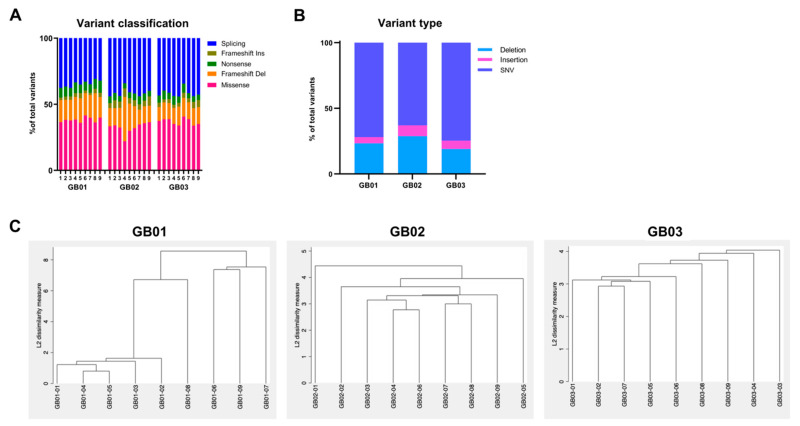
Rare variants. (**A**) Proportion of variant classification, plotted as the percentage of the total number of detected rare variants. (**B**) Proportion of mutation type, plotted as the percentage of the total number of detected rare variants. (**C**) Dendrograms of hierarchical cluster analysis of rare mutations of tumor regions. Dendrograms graphically present the information concerning which tumor regions are grouped together at various levels of (dis)similarity. At the bottom of the dendrogram, each tumor region is considered its own cluster. The height of the vertical lines and the range of the (dis)similarity axis give visual clues about the strength of the clustering. Long vertical lines indicate more distinct separation between the groups. Long vertical lines at the top of the dendrogram indicate that the groups represented by those lines are well separated from one another. Shorter lines indicate groups that are not as distinct.

The variant classification plot in Figure 4A shows the number and classification of variants in each tumor region, providing percentages of missense, nonsense, splice-site, and frame shift mutations (both insertion and deletion) out of the total number of mutations.

To assess the similarities and differences between each of the intra-tumoral regions, as well as between different tumors, we performed hierarchical clustering on the rare mutations (Figure 4C). The hierarchical analysis generates three different clustering profiles, one per tumor, based on the dissimilarity of the 9 intra-tumoral portions due to the presence/absence of rare tumor mutations. The length of the vertical lines in each dendogram is proportional to the differences in rare mutations among individual tumor regions or among groups of regions. Tumor GB01 generates a dendrogram different from the other two tumors. GB01 is divided into two main groups, GB01-06/07/09 on one side and GB01-01–05/08 on the other side. In the second group GB01–08 differs from the other 4 regions (01–05) which are more similar to each other. In contrast, the intra-tumoral regions of GB02 appear to have profound dissimilarities with each other, and the cluster shows a progressive subdivision into gradually smaller subgroups with increasingly similar regions. Furthermore, in GB03, all nine intra-tumoral portions show great dissimilarity from each other. With the exception of regions GB03–02 and G03–07, which appear to have more similarity, the other regions show more independent profiles.

**Figure 5:**
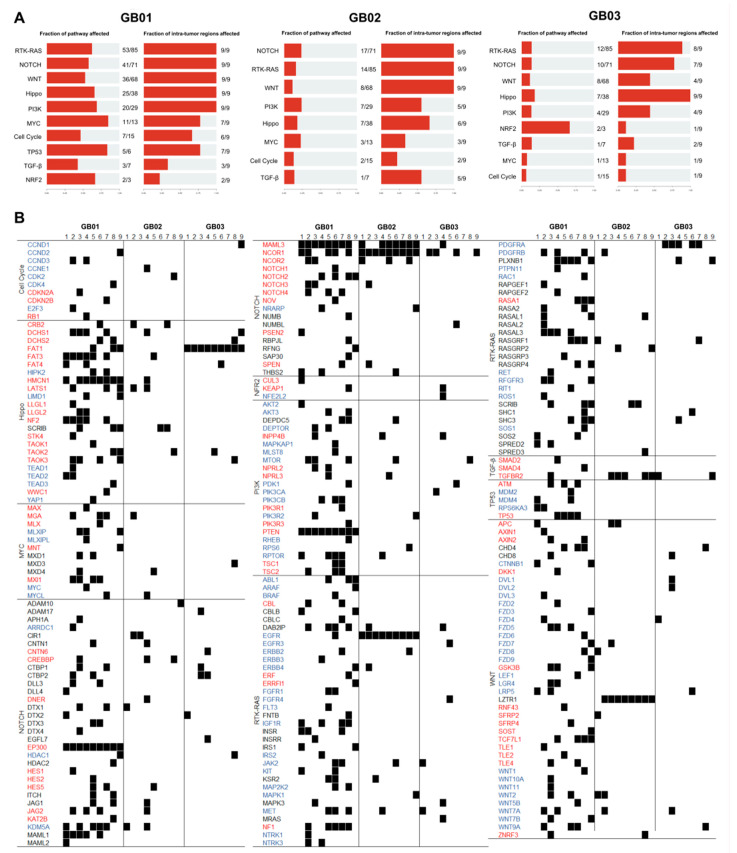
Oncogenic pathways. (**A**) Pathway alteration frequencies. Fraction of mutated genes for each pathway and fraction of tumor regions with mutated genes for that pathway. (**B**) Detail of mutated genes in their respective altered pathways for each of the 27 tumor regions. Black square indicates the presence of a rare mutation. Tumor-suppressor genes are in red and oncogenes are in blue.

Using MafTools’ OncogenicPathways [21], we performed an enrichment of known oncogenic signaling pathways [30]. We then studied rare somatic alterations in the ten canonical pathways: cell cycle, Hippo, MYC, NOTCH, NRF2, PI-3-Kinase/AKT, RTK-RAS, TGFβ signaling, p53 and β-catenin/WNT. For each tumor, we calculated the fraction of altered genes belonging to each pathway and the fraction of intra-tumor regions with alterations in genes of that specific pathway (Figure 5A).

As shown in Figure 5A, in GB01, the p53 and MYC pathways with affected pathway fraction of 0.8 (5/6 genes and 11/13, respectively) were the most affected, followed by PI3K (20/29), NRF2 (2/3), RTK-RAS (53/85), Hippo (25/38), NOTCH (41/71), WNT (36/68), cell cycle (7/15), and TGF-β (3/7). The RTK-RAS, NOTCH, WNT, Hippo, and PI3K pathways were affected in nine/nine regions in GB01 (Figure 5A). In GB02, the most affected pathways were PI3K (7/29), NOTCH (17/71), and MYC (3/13). Rare mutations were also found in genes belonging to the Hippo (7/38), RTK-RAS (18/85), TGF-β (1/7), Cell Cycle (2/15), and WNT (8/68) pathways. The RTK-RAS, NOTCH, and WNT pathways were mutated in nine/nine regions in GB02. In GB03, the NRF2 pathway with affected fraction of 0.7 (two/three genes) was the most affected, followed by Hippo (7/38), TGF-β (1/7), RTK-RAS (12/85), NOTCH (10/71), PI3K (4/29), WNT (8/68), MYC (1/13), and Cell Cycle (1/15). The Hippo pathway is the only pathway mutated in nine/nine regions in GB03.

To analyze in detail the involvement of these pathways in the various tumor regions, in Figure 5B we list all existing genes within each pathway and highlight with a black square denoting the mutated genes. Among the genes in Figure 5B there are tumor suppressor genes (red font) and oncogenes (blue font). There are mutated tumor suppressor genes in all (nine/nine) tumor portions, such as *EP300* and *PTEN* in GB01, *NCOR2* in GB02, and *FAT1* in GB03. While for oncogenes, the GB02 tumor has (nine/nine) *EGFR* mutations in all its portions. The three tumors have several mutated genes in common in at least one of their portions: *DCHS1* and *TAOK2* (Hippo), *MET*, *JAK2*, *DAB2IP*, and *RASGRF1* (RTK-RAS), *MTOR* and *INPP4B* (PI3K), *NCOR1* and *MAML3* (NOTCH), *TGFBR2* (TGF-β), and *WNT7a* (WNT). Figure 5B reveals that many genes (including tumor suppressor genes or oncogenes) are mutated in only one of the nine tumor portions.

### 3.6. Phylogenetic and Clonal Evolution

To further select potential disease-related genes, an additional filtering step was added to rare variants. Rare mutations in genes already described and annotated in COSMIC in GB tumors were considered for these two analyses.

**Figure 6:**
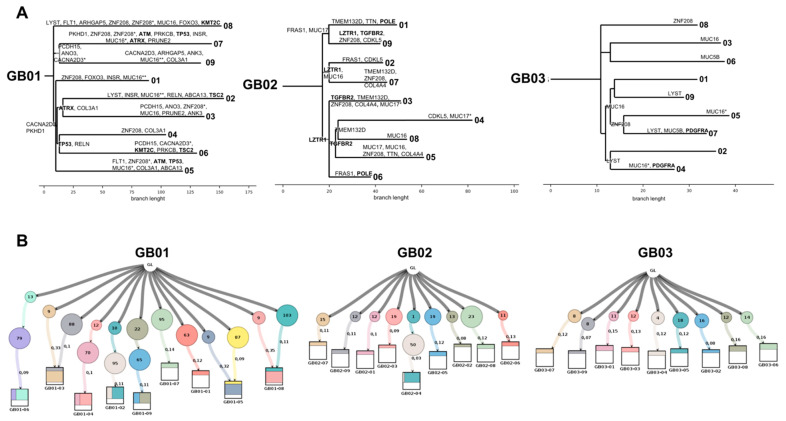
Tumor phylogeny and clonal evolution. (**A**) Phylogenetic trees. For each sample, a rooted tree was created whose leaf nodes are tumor regions. The length of the branches is equal to the number of mutations. Genes annotated on the tree are those with which the system has distinguished (through the absence or presence of specific mutations) and divided the different leaf branches representing the 9 tumor portions. Genes in bold are driver genes. (**B**) Clonal evolution lineage tree and sample composition. The lineage tree was built based on the constraint network using Lineage Inference for Cancer Heterogeneity and Evolution (LICHeE). Each node (circle) represents a subpopulation of GB cells. All nodes arose from a single hypothetical clone called Germline (GL), representing the genetic architecture of normal tissue from the same patient. Numbers within the circles indicate the number of nucleotide variants shared by the cluster; numbers outside the circles show the average variant allele frequency (VAF) of the variants in each cluster. Squares represent each individual tumor region, with colored rectangles indicating the cell fraction represented by the clonal cluster.

We used phylogenetic tree inference (PTI) [31] to infer the rooted phylogenetic tree between different regions of the same tumor. Once the mutations are identified for all samples and the number of shared mutations are defined, the system forms the trunk of the tree. Then, through the unique mutations of individual regions and those shared by two or more tumor regions, the PTI finds the optimal branch division until all tumor regions reach the leaf nodes (Figure 6A and Table S2).

The trees are built on the presence/absence of mutations; leaves from the same branch share some mutations but differ in others. Thus, each leaf (tumor region) will be unique due to the presence of a specific combination of mutations. By following the early (mutations shared between regions, present in the trunk/branches) or late (mutation at the level of the leaves) occurrence, it will be possible to reconstruct the mutational history of these tumors. Each trunk/branch/leaf shown in Figure 6A has a length dictated by the number of mutations present. The distinction between intra-tumoral regions is dictated only by the mutations in the genes that appear on the phylogenetic tree structure in Figure 6A. Among these mutated genes, we also found driver genes (in bold in Figure 6A): KMT2C, ATM, ATRX, and TSC2 in GB01; LZTR1, TGFBR2, and POLE in GB02; and PDGFRA in GB03. Some driver gene mutations are early mutations because they are present in branches shared by more than one tumor region and thus arose earlier than others exclusive to the regions. Among these, we find *ATRX* and *TP53* in GB01 and, *LZTR1* and *TGFBR2* in GB02.

To gain insight into the evolutionary status of these three GB cases and to reconstruct multi-sample tumor phylogenies and decompose tumor subclones, we used LICHeE (Lineage Inference for Cancer Heterogeneity and Evolution). This system uses the variant allele frequencies of rare somatic mutations to reconstruct multi-sample cell lineage trees and infer the subclonal composition of the samples. The evolution trees and subclonal composition of GB tumors are shown in Figure 6B. The complete list of representative mutations in each clonal subpopulation is in Table S3.

For all three GBs, each tumor region is characterized by at least one clonal subpopulation exclusive to that region (colored rectangles within the square representing the tree leaf). In some regions (02, 04, 06 and 09 in GB01 and 04 in GB02) we note that two clonal subpopulations coexist, one within the other. As an example, in the case of GB01-06 (Figure 6B), 13 mutations are present in the tumor region with a VAF of 0.33, corresponding to their presence in 33% of the cells in the region, which in turn has a percentage of cells (9%) characterized by the presence of other 79 mutations with VAF 0.09. Other regions instead (03, 05 and 08 in GB01) are characterized by the co-presence of two distinct subclones. In the case of GB1-03 (Figure 6A), the tumor region is composed of 3 clonal subpopulations: 33% of cells (VAF 0.3) are characterized by nine mutations, 10% (VAF 0.1) by 88 mutations, and the rest by cells sharing the same mutations and VAF with the other regions.

## 4. Discussion

GB is the most frequent brain tumor in adults and is characterized by an invariably fatal prognosis [32]. With optimal treatment, the median survival of patients is just over 1 year and only 18% survive two years [33]. GB is considered one of the most feared of all human diseases, both because of the lack of a cure and the associated loss of cognitive function as part of the disease process [27]. Currently, there are few biomarkers of favorable prognosis and consequently few therapies that strongly influence disease outcome. This is primarily due to the fact that the extreme heterogeneity of these tumors makes therapies increasingly challenging.

Along with inter-tumor heterogeneity, intra-tumor heterogeneity represents a pivotal area of investigation of GB. Several genomic studies have highlighted this heterogeneity by demonstrating a difference in somatic alterations, expression subtypes, and epigenetic modifications between different GBs and within the same tumor [13,27,34,35,36,37,38,39,40]. Other studies have attempted to go even further by specifically examining regional heterogeneity between multiple sectors of the same primary GB, sometimes also coupled to relapsed tumor, through whole exome and transcriptome sequencing [2,3,41,42,43]. Notably, multiregional exome sequencing studies of primary GB have been conducted on up to four tumor portions [2,41,42].

In this study, we aimed to further improve the analysis by going into even more detail in tumor heterogeneity. Therefore, we increased the number of tumor portions to be analyzed by choosing nine regions and analyzing them by whole exome sequencing. We also sought to ensure cohort homogeneity by selecting only GB IDH-WT without 1p/19q codeletion and patients of the same sex (women), with similar age and clinical course. Through a multiregional study of tumors from post-surgical samples, we aim to increase our understanding of tumor evolution and highlight the importance of tumor sampling from spatially distinct regions to avoid misinterpretation of genomic data from a single sample collection. Despite the well-known difficulties in collecting multiple samples of a glioblastoma in surgical procedures, the goal of deepening the understanding of tumor heterogeneity is also useful in devising potential new solutions to address the complexities of tumor heterogeneity in the face of the reality of therapeutic decisions based on limited access to tumor tissue.

All three of our samples are characterized by an extremely high level of DNA copy number alteration, as shown in Figure 2A,B. Several copy number variations, reported within and among the three tumors, commonly characterize primary IDH-WT GB, such as: deletion of chromosome 9 and 10 and amplification of chromosome 7 [44,45]. However, there are intra-tumoral differences in gain and loss at loci 9q, 7p, and 7q. Other observed alterations are deletion of 13q and amplification of 19q, both previously described in GB [46], both with intra-tumoral differences. In many other chromosomal loci (15q, 19p, 20q, 22q, and entire chromosomes 3, 8, 16, and 17), alterations, both deletions and amplifications, are present and are reflected quite unevenly across tumor regions.

A molecular classification of GB performed by The Cancer Genome Atlas (TCGA) identified four molecular subgroups with presumed prognostic significance [27]. The subgroups of GB described by TCGA, namely classical, neural, proneural, and mesenchymal, were identified and classified based on transcriptional profiles and supplemented with mutational and DNA copy number alteration profiles [27]. We relied on this classification system to define the molecular subtype of our tumor regions by correlating changes in DNA copy number. As displayed in Figure 2C,D, for all three samples, within the same tumor, more than one molecular subtype was correlated and identified. This confirms the findings of Sottoriva and colleagues [3], who through gene expression analysis, have observed the coexistence of different molecular subtypes within single GB tumors. From a mutational perspective, we first looked for somatic mutations in genes known to be mutated in GB. Although some genes (*APC*, *ATM*, *ATR*, *FAT1*, *EGFR*, *ERBB3*, *BRIP1*, *PTEN*, *IK*, *MSH6*, *PIK3CA* and *TP53*) were found to be mutated in all regions of the same tumor, the type of alteration is not always the same in all nine regions. In addition, there are also several genes that carry mutations in only one or two of the nine regions of the same tumor (*ARID2*, *BAD*, *BCL2*, *BRAF*, *CBL*, *CDKN2B*, *FBXW7*, *KMT2D*, *MDM2*, *MET*, *MYC*, *PIK3CA*, *PTEN*, *SF3B1*, *SOX2*, *TERT* and *TP53*). All of these variants were then ranked for their predicted pathogenicity, and a total of 59 variants in 18 genes were reported as pathogenic or probably pathogenic. None of these mutations are shared by all regions of the three tumors. Of these 59 variants found, 86% are mutations that affect only a single region in the nine within the tumor, meaning that the mutation is present in only 11% of the cells of the entire tumor. Twenty-six variants are known COSMIC mutations, and among them, two variants have already been described in GB [47,48]: *EGFR*^Arg108Lys^ present in all regions of GB02 and *KMT2D*^Arg2830Ter^ in region 07 of GB01.

We then further performed several analyses selecting the rare somatic mutations found in our samples. We first performed an unsupervised analysis to group the nine regions of each tumor according to the mutations they carried. In all three tumors, the tumor regions appear to be very dissimilar to each other, except for GB01, where we note four regions (01–05) that are more closely related to each other. The regions of tumors GB02 and GB03 cluster in distinct ways; in GB02, the cluster shows a gradual division into smaller and smaller groups with increasingly similar regions to each other; in GB03, all nine intra-tumoral portions show great dissimilarity to each other. In each tumor, however, the regions show highly independent profiles.

Next, we investigated how rare mutations might affect the 10 canonical cancer molecular pathways: cell cycle, Hippo, MYC, NOTCH, NRF2, PI-3-Kinase/AKT, RTK-RAS, TGFβ signaling, p53, and β-catenin/WNT. Within each of these pathways, we looked in detail at the mutated genes and alterations that occur in each tumor region. As reported in Figure 5B, there are both suppressor genes and tumor oncogenes mutated in all nine/nine parts of the tumor, such as *EP300* and *NCOR2* (NOTCH), *PTEN* (PI3K), *EGFR* (RTK-RAS) and *FAT1* (Hippo). Other genes belonging to these oncogenic pathways were found mutated in all three tumors in at least one of their regions: *DCHS1* and *TAOK2* (Hippo), *MET*, *JAK2*, *DAB2IP* and *RASGRF1* (RTK-RAS), *MTOR* and *INPP4B* (PI3K), *NCOR1* and *MAML3* (NOTCH), *TGFBR2* (TGF-β) and *WNT7a* (WNT). Most importantly, many tumor suppressor genes or oncogenes are mutated in only one of the nine tumor portions.

From the list of mutated genes and pathways that emerges from our analyses, it is important to underline how our results confirm data already reported in the literature. In fact, it is well known as genetic alterations in GB typically deregulate pathways involving the tumor suppressors p53 (87%), RB (78%), and receptor-tyrosine kinase (RTK)/RAS/PI3K (88%) [49]. The PI3K/Akt/mTOR signaling pathway after EGFR activation is one of the most significant signaling pathways in cancer cells, and as clinical research has revealed, mutations in both *EGFR* and *PTEN* would lead to continuous activation of the signaling pathway, thus contributing to tumorigenesis and resistance to therapy [50]. *INPP4B* has been identified as a tumor suppressor often associated with *PTEN* in several cancers, and loss of INPP4B protein has also been correlated with reduced patient survival [51]. Mutations in *MET*, as well as the dysregulation of other regulators of crosstalk with MET signaling pathways in glioma, have also been previously identified and associated with proliferation, survival, migration, invasion, angiogenesis, stem cell characteristics, therapeutic resistance, and glioblastoma recurrence [52]. *JAK2*, in the RTK-RAS pathway has also garnered significant interest in the past as a key driver of cancer cell survival, proliferation and invasion in GB [53].

The Notch pathway has already been described in glioblastoma tumorigenesis [54] and correlated with GB development and proliferation as well as with its prognosis [55]. Specifically, EP300 and NCOR2 expression levels were correlated with survival and therapeutic response in GB [56]. In cancer, *EP300* has been previously reported to target both mutations and structural alterations [57], which could be an important contributor to malignant transformation [58,59,60]. Growing evidence has demonstrated the role of Hippo signaling in cancer biology, and its alteration has been closely linked to tumorigenesis in many different cancers, also playing a key role in glioma development, including cell proliferation, apoptosis, and invasion [61,62]. *FAT1* mutations in GB have already been reported, and studies strongly suggest that members of the FAT gene family are important players in cancer development [63,64,65].

As a concluding analysis, we aimed to reconstruct phylogenetic and clonal evolution trees (Figure 6A,B), either by accounting for the presence/absence of a mutation in different tumor regions and by decomposing GB into tumor subclones. We performed this analysis using rare mutations of genes already described and annotated in COSMIC in GB tumors. Although we find early mutations, including those of driver genes (*ATRX*, *TP53*, *LZTR1* and *TGFBR2*), present in branches shared by more than one tumor region, the results of this analysis demonstrate a high degree of intra-tumoral heterogeneity in the three patients, with private leaf node mutations dominating the mutational landscape. By analyzing the allele frequencies of rare somatic mutation variants to reconstruct multi-sample cell lineage trees and infer the subclonal composition of the samples, we found that each tumor region is characterized by at least one clonal subpopulation exclusive to that region. Indeed, some regions show two clonal subpopulations, one within the other, while others are characterized by the co-presence of two distinct subclones.

In this work, we conducted most of the mutational investigations focusing on altered genes already described in GB; however, the majority of the variations found have not yet been annotated. The large number of mutations identified in our samples may therefore also include mutations that have not been functionally characterized in the current literature. Further future studies are certainly needed to investigate their functional roles.

The high level of heterogeneity of these three GBs is shown both at the mutational level, in somatic mutations, even rare ones, in DNA copies and also in the chronology of mutational evolution and in the subclonal composition of individual tumor regions. As never reported before, by increasing the regional subdivision of the tumor, up to 9 portions, the intra-tumor heterogeneity remains highly represented. All this suggests that most mutations are transient mutations, and that each intra-tumoral region is characterized by its own alterations. This seems in agreement with what was previously discussed by Sottoriva [3]. In particular, we can speculate that there are multiple clones with different fitness co-existing within GB.

The study and understanding of these patterns of heterogeneity could then be used to stratify individual patients and select an appropriate therapeutic strategy. To do this, however, multiple sampling is strictly necessary, as it is highly unlikely that a single biopsy could represent the complete set of mutations present underestimating the landscape of all alterations present. There are also hypotheses for which characterization of GB intra-tumor heterogeneity using single cell sequencing technology would be necessary [66]. Heterogeneity among tumor cells does not arise only as a result of molecular and genetic changes but is also influenced by different microenvironments within the tumor and reversible changes in cellular properties [67,68]. GB is composed of an interactive network of neoplastic and non-neoplastic cells, characterized by cell-cell interactions and connections of cellular compartments, creating a complex microenvironment that constantly gives signals, activating cell migration and ultimately developing permissive niches that promote tumor cell survival and proliferation [69,70] and influence therapeutic response [70,71,72]. Correlating key histological features of GB, different tumor microenvironments, and intracranial tumor locations to mutational patterns would allow for a greater and more complete understanding of tumor heterogeneity.

As Spiteri [43] also suggests, not only primary tumor should be studied, but residual disease in relapsed GB cases is equally important to be investigated to understand how treatment-resistant disease develops and to further investigate tumor evolution. It would also be important and useful to develop improvements during tumor surgery such as harvesting the subventricular zone and infiltrative margin, which is currently not possible due to the lack of reliable tumor cell markers [43].

## 5. Conclusions

In conclusion, our study is the first to use multiregional WES in such a large number of portions (nine tumor regions per tumor) to study the tumor heterogeneity. Although the sample size of the current study is relatively small (three primary IDH-WT GBs), our data provided additional insights into intra-tumoral heterogeneity, painting a still incomplete picture that warrants further investigation. Despite therapeutic opportunities for GB remain limited, any continued effort to study and understand tumor heterogeneity may help find a cure for this devastating disease.

## Figures and Tables

**Table 1:** Patient clinical and demographic data. Age, age at diagnosis. MGMT (O-6-methylguanine-DNA methyltransferase), percentage of MGMT promoter methylation. Region, tumor location and surgical resection area. Therapy, Radiotherapy (RT) and chemotherapy with temozolomide (TMZ). RFS, recurrence-free survival. OS, overall survival.

Tumor	Sex	Age	MGMT	Region	Therapy	RFS	OS
GB01	F	55	8%	Left peritrigonal area	TMZ concurrent with RT followed by adjuvant TMZ	13 months	20 months
GB02	F	74	56%	Right parietal lobe	RT	nd	nd (>6 months)
GB03	F	58	<7%	Right frontal-temporal-parietal area	TMZ concurrent with RT followed by adjuvant TMZ	16 months	20 months

**Table 2:** Enrichment of pathogenic variants in GB01, GB02, and GB03 somatic mutations. Gene, mutated gene. Variant, amino acid change or base substitution. Tumor (regions), tumor regions in which that mutation was detected. Genotype, heterozygous (het) or homozygous (hom) mutation. Type, type of genetic mutation. COSMIC, mutation already noted in the Catalogue of Somatic Mutations in Cancer (COSMIC). Described in CNS, variants that have already been described in brain tumor and annotated in COSMIC. Varsome, functional prediction of each variant classified using American College of Medical Genetics and Genomics (ACMG) rules.

Gene	Variant	Tumor (Regions)	Genotype	Type	Cosmic	Described in CNS	VARSOME
*APC*	Gln163Lys	GB01-04	het	Missense			Likely Pathogenic
*ATM*	Ser421Ter	GB01 (01, 02, 03, 04, 06, 07), GB02 (03, 07, 08)	het	Nonsense	COSM4165637		Pathogenic
*ATR*	Glu359Ter	GB02–05	het	Nonsense			Pathogenic
	Arg1951Ter	GB03–06	het	Nonsense	COSM6933109		Pathogenic
	Gln264Ter	GB01–03	het	Nonsense			Pathogenic
	Trp2094Ter	GB01–04	het	Nonsense			Pathogenic
*BRAF*	Arg271Ser	GB02–09	het	Missense			Likely Pathogenic
	Glu375Ter	GB01 (05)	het	Nonsense	COSM5702658		Pathogenic
*EGFR*	Arg108Lys	GB02 (all)	het	Missense	COSM21683	Y	Likely Pathogenic
	Thr273Pro	GB02–02	het	Missense			Likely Pathogenic
*ERBB3*	Lys695ArgfsTer15	GB01–04	het	Frameshift			Pathogenic
	Leu12Ter	GB01–01	het	Nonsense			Pathogenic
	c.2840-2A > G	GB01–02	het	Splice Site			Pathogenic
*KMT2D*	Gln3370SerfsTer22	GB03–03	het	Frameshift			Pathogenic
	Arg2830Ter	GB01–07	het	Nonsense	COSM220674	Y	Pathogenic
*MET*	Thr230ArgfsTer33	GB01–05	het	Frameshift			Pathogenic
	Asp449HisfsTer4	GB01–08	het	Frameshift			Pathogenic
*MSH6*	Ala1302GlufsTer25	GB01–09	het	Frameshift			Pathogenic
	Phe115LeufsTer34	GB01–09	het	Frameshift			Pathogenic
	Phe1088SerfsTer2	GB01–09, GB02 (05, 06), GB03 (08)	het	Frameshift			Pathogenic
*MYC*	Thr73Pro	GB01–01	het	Missense			Likely Pathogenic
*NOTCH1*	Val1229LeufsTer216	GB01–03	het	Frameshift			Pathogenic
	His2275ProfsTer20	GB01–08	het	Frameshift			Pathogenic
	Met2237CysfsTer11	GB03–02	het	Frameshift			Pathogenic
	c.1556-2A > C	GB02 (02, 05)	het	Splice Site			Pathogenic
	c.4587-2A > C	GB03 (08)	het	Splice Site			Pathogenic
	Gly1339AlafsTer106	GB03–06	het	Splice Site			Pathogenic
*PIK3CA*	Ser1003Leu	GB01–01	het	Missense	COSM5700983		Likely Pathogenic
	Leu649Ile	GB01–03	het	Missense			Likely Pathogenic
	Ile391Lys	GB01–08	het	Missense			Likely Pathogenic
	Phe667Cys	GB02–01	het	Missense			Likely Pathogenic
	Cys838Trp	GB03–05	het	Missense			Likely Pathogenic
	Met1043Ile	GB03 (all)	het	Missense	COSM773		Pathogenic
	c.1540-1G > T	GB03–02	het	Splice Site			Pathogenic
*PTEN*	Asp52Asn	GB01 (all)	het	Missense	COSM5059		Likely Pathogenic
	Trp274Cys	GB02–06	het	Missense			Likely Pathogenic
	His272Gln	GB02–07	het	Missense			Pathogenic
	c.1027-2delA	GB01–07	het	Splice Site			Pathogenic
*RB1*	Leu337TrpfsTer12	GB01–04	het	Frameshift			Pathogenic
	Leu683Pro	GB01–01	het	Missense			Likely Pathogenic
	Leu448Ile	GB01–02	het	Missense			Likely Pathogenic
	Leu400Met	GB01–04	het	Missense			Likely Pathogenic
	His483Asn	GB01–05	het	Missense			Likely Pathogenic
	Ile711Leu	GB02–07	het	Missense			Likely Pathogenic
	Glu465Ter	GB01–05	het	Nonsense	COSM6936008		Pathogenic
	Tyr446Ter	GB02–02	het	Nonsense			Pathogenic
	Ser474Ile	GB01 (01–07) GB02 (03, 04, 08, 09)	het	Splice Site	COSM4807851		Pathogenic
	c.1421 + 1G > T	GB01 (02, 03, 05), GB02 (03)	het	Splice Site			Pathogenic
*SF3B1*	Tyr623His	GB01–01	het	Missense			Likely Pathogenic
	Ile704Met	GB03–03	het	Missense			Likely Pathogenic
	Glu62Ter	GB03–03	het	Nonsense			Pathogenic
	Gln1252His	GB02–03	het	Splice Site			Likely Pathogenic
	Gln1252Ter	GB01–09	het	Splice Site			Pathogenic
*SOX2*	Arg57AlafsTer46	GB01–08	het	Frameshift			Pathogenic
	Ala94Asp	GB01–08	het	Missense			Likely Pathogenic
*TERT*	Ile1046Ter	GB01–09	het	Frameshift			Pathogenic
	c.2582 + 2T > G	GB02–08	het	Splice Site			Pathogenic
*TP53*	Tyr103Ser	GB02–09	het	Missense			Likely Pathogenic
	Gln144Ter	GB01–01	het	Nonsense			Pathogenic

## Data Availability

The nucleic acid sequences were deposited in the The European Genome-phenome Archive (EGA): https://ega-archive.org, accessed on 8 March 2021 (submission ID: EGAD00001007063).

## Supplementary Materials

The following are available online at https://www.mdpi.com/article/10.3390/cancers13092044/s1. Table S1: Sequencing run information for each tumor region. Mapped reads, total number and percentage of reads mapped inside target regions; Coverage, mean of the coverage depth; Quality, mean mapping quality of the mapped reads; CG percentage, GC percentage in the mapped reads, Table S2: Binary matrix output from PTI analysis for GB01, GB02 and GB03. The first two columns record unique gene and mutation IDs. The middle columns record whether a single tumor portion (leaf) has a certain mutation or two/more tumor regions (trunk or branches) share it. 1 = mutation, 0 = no mutation. The last column notes whether the genes involved in the mutation are genes drivers, Table S3: Representative mutations in each subclonal population. Sample, tumor; Region, tumor region; Size, number of mutations in that specific subpopulation; VAF, variant allele frequency of representative mutations; Gene, mutated genes; Mutation, mutation detail; AA change, amino acid change.

## Abbreviations

CNV	Copy number variation
GB	Glioblastoma
GCS	Global score
MAF	Minor allele frequency
PTI	Phylogenetic tree inference
SNV	Single-nucleotide variant
VAF	Variant allele frequency
WES	Whole-Exome Sequencing
